# SRPX2 attenuated oxygen–glucose deprivation and reperfusion-induced injury in cardiomyocytes via alleviating endoplasmic reticulum stress-induced apoptosis through targeting PI3K/Akt/mTOR axis

**DOI:** 10.1515/biol-2022-0513

**Published:** 2022-11-14

**Authors:** Zhiyuan Sun, Xin Gao

**Affiliations:** Department of Cardiovascular, Tianjin Fifth Central Hospital, Tianjin 300450, China; Department of Cardiology, Affiliated Hospital of Integrated Traditional Chinese and Western Medicine, Nanjing University of Chinese Medicine, No. 100, Cross Street, Hongshan Road, Nanjing City, Jiangsu Province 210028, China

**Keywords:** myocardial infraction, Sushi repeats contain the protein X-Linked 2, myocardial ischemia/reperfusion, ER stress, PI3K/Akt/mTOR pathway

## Abstract

Myocardial infraction (MI) is the leading cause of high morbidity and mortality worldwide. It was still urgently needed to find new and effective drugs for MI treatment by the use of myocardial ischemia/reperfusion (I/R) model. Sushi repeats contain the protein X-Linked 2 (SRPX2), which regulates a variety of important cell functions. However, its possible role in myocardial I/R and the progression of MI is still unclear. In this study, we investigated the role of SRPX2 in myocardial I/R. SRPX2 showed low expression in IR rats and H9C2 cells induced by oxygen–glucose deprivation/reperfusion (OGD/R). SRPX2 could increase OGD/R-induced H9C2 cell survival. In addition, SRPX2 suppressed the apoptosis of OGD/R-induced H9C2 cells. Furthermore, we found that SRPX2 could inhibit ER stress induced by OGD/R in H9C2 cells. Mechanically, we found that SRPX2 suppressed the PI3K/Akt/mTOR pathway, thus attenuating OGD/R -induced injury in H9C2 cells. Therefore, SRPX2 has the potential to serve as a target for MI treatment.

## 1 Introduction

Myocardial infraction (MI) is the leading cause of high morbidity and mortality worldwide [1]. The mechanism of myocardial repair is complex. At present, the treatment of MI can only relieve the symptoms and cannot solve the problem of root myocardial cell loss [2,3]. Therefore, the pathogenesis of this disease still deserves further investigation [4]. Myocardial ischemia/reperfusion (I/R) injury is a serious pathological condition associated with myocardial apoptosis [5–7]. Myocardial ischemia–reperfusion is often used as a disease model to explore new treatments or drugs for MI [8]. In recent years, some innovative methods have been developed for cardiomyocyte evaluation and MI treatment, such as scanning electrochemical microscopy-based methods, which evaluate the REDOX activity of human myocardial-derived mesenchymal stem cells by scanning electrochemical microscopy [9]. However, it was still urgently needed to find new and effective drugs for MI treatment by using myocardial I/R model.

Sushi repeats contain the protein X-Linked 2 (SRPX2), a novel chondroitin sulfate proteoglycan originally identified in leukemia cells, which regulates a variety of important cell functions, such as cell growth, migration, and adhesion [10]. Its role in tumorigenesis has been widely revealed. For example, SRPX2 increased the proliferation and migration of osteosarcoma cells by regulating Hippo pathway, thus promoting the poor prognosis of patients [11]. In addition, SRPX2 increased the proliferation and suppressed apoptosis of colon cancer cells by activating Wnt/β-catenin axis [12]. SRPX2 can also promote the malignant progression of pancreatic cancer, lung cancer, and esophageal cancer [13].

Notably, SRPX2 was also a ligand of a novel hypothalamic protein and urokinase-type plasminogen activator receptor, which is essential for the proteolysis of the extracellular matrix following the process of brain injury [14]. It was also found that traumatic brain injury (TBI) induced a decrease in SRPX2 expression and a decrease in the number of bilateral SRPX2 immunoreactive neurons [15]. Therefore, a decrease of SRPX2 levels may be a candidate biomarker of hypothalamic injury [16]. Among the differentially expressed genes (DEGs) revealed by RNA sequencing in MI myocardium, SRPX2 was one of the significantly downregulated DEGs [17]. Importantly, SRPX2 could also activate the PI3K/Akt/mTOR pathway, thereby inhibiting ER stress-stimulated apoptosis and further improving I/R-induced cardiomyocyte injury [18].

In this study, we investigated the role of SRPX2 in myocardial I/R cell model. Our results indicated that the expression of SRPX2 was significantly decreased. Overexpression of SRPX2 could increase the survival of H9C2 cells, and suppress the ER-stress-induced apoptosis of H9C2 cells induced by oxygen–glucose deprivation/reperfusion (OGD/R) through PI3K/Akt/mTOR pathway.

## 2 Materials and methods

### 2.1 Cell culture and treatment

Rat embryonic heart cells H9C2 were purchased from American Type Culture Collection (ATCC, Rockville, MD) and incubated in serum (10%), which constituted the Dulbecco’s modified Eagle’s medium (DMEM) complete medium at 37°C in culturing hood supplied with 5% CO_2_.

Human AC16 cardiomyocyte cell line was obtained from ATCC and were cultured in DMEM-F-12 medium supplemented with 12.5% fetal calf serum at 37°C in culturing hood supplied with 5% CO_2_.

For construction of the OGD/R model, H9C2 and AC16 cells were washed with PBS and cultured with glucose-free DMEM in a hypoxic culturing hood inflated with 94% N_2_, 5% CO_2_, and 1% O_2_ at 37°C for oxygen and glucose deprivation. Then cells were maintained in complete DMEM and cultured under normoxic conditions for 24 h. Control cells were left untreated.

### 2.2 Cell viability

Cells were plated into 96-well plates at the density of 3 × 10^3^ cells/well. Cells were added with 3-(4,5)-dimethylthiahiazo (-*z*-y1)-3,5-di-phenytetrazoliumbromide (MTT) for cell viability detection followed by washing with PBS. After incubation for 4 h, the absorbance was assessed at 490 nm.

### 2.3 Lactate dehydrogenase (LDH) detection

LDH level was detected with an LDH cytotoxicity detection kit (Roche, Basel, Switzerland). Briefly, the culture supernatant in each group was collected and LDH levels in the culture medium were obtained at 490 nm with a microplate reader.

### 2.4 Terminal deoxynucleotidyl transferase dUTP nick-end labeling (TUNEL) assay

Cell apoptosis was detected with TUNEL assay kit following its protocols (Roche Diagnostics). Briefly, cells were fixed with 10% PFA, permeabilized, and incubated with TUNEL reaction solution in a wet dark box for 1 h at 37°C. Nuclei were counterstained with DAPI and images were captured under a microscope.

### 2.5 Western blotting

Cell lysates were prepared using RIPA buffer (Beyotime, China). After centrifugation, BCA protein assay kit (Beyotime Biotechnology) was used for protein concentration detection. Then proteins were separated by 10% SDS-PAGE, transferred onto PVDF membranes. After blocking with 5% BSA in tris-buffered saline for 1 h, the membranes were incubated with Bax (1:1,000, Abcam), bcl-2 (1:1,000, Abcam), Cleaved Caspase-3 (1:1,000, Abcam), Caspase-3 (1:1,000, Abcam), GRP-78 (1:1,000, Abcam), XBP-1 (1:1,000, Abcam), ATF-6 (1:1,000, Abcam), ATF-4 (1:1,000, Abcam), CHOP (1:1,000, Abcam), p-PI3K (1:1,000, Abcam), PI3K (1:1,000, Abcam), p-AKT (1:1,000, Abcam), AKT (1:1,000, Abcam), p-mTOR (1:1,000, Abcam), mTOR (1:1,000, Abcam), and anti-β-actin (1:10,000; Abcam) antibodies at 4°C overnight. Membranes were resolved in HRP-conjugated secondary antibodies at 1:1,000 for 2 h followed by washing for 15 min with TBST. The signals were visualized using ECL detection kit.

### 2.6 Statistical analysis

Data were analyzed statistically by the Student’s *t*-test. Graphs were obtained using GraphPad Prism 6.0 software (San Diego, CA, USA). Three repeats were performed for each experiment.

## 3 Results

### 3.1 SRPX2 increases OGD/R-induced cell viability in H9C2 and AC16 cells

**Figure 1:**
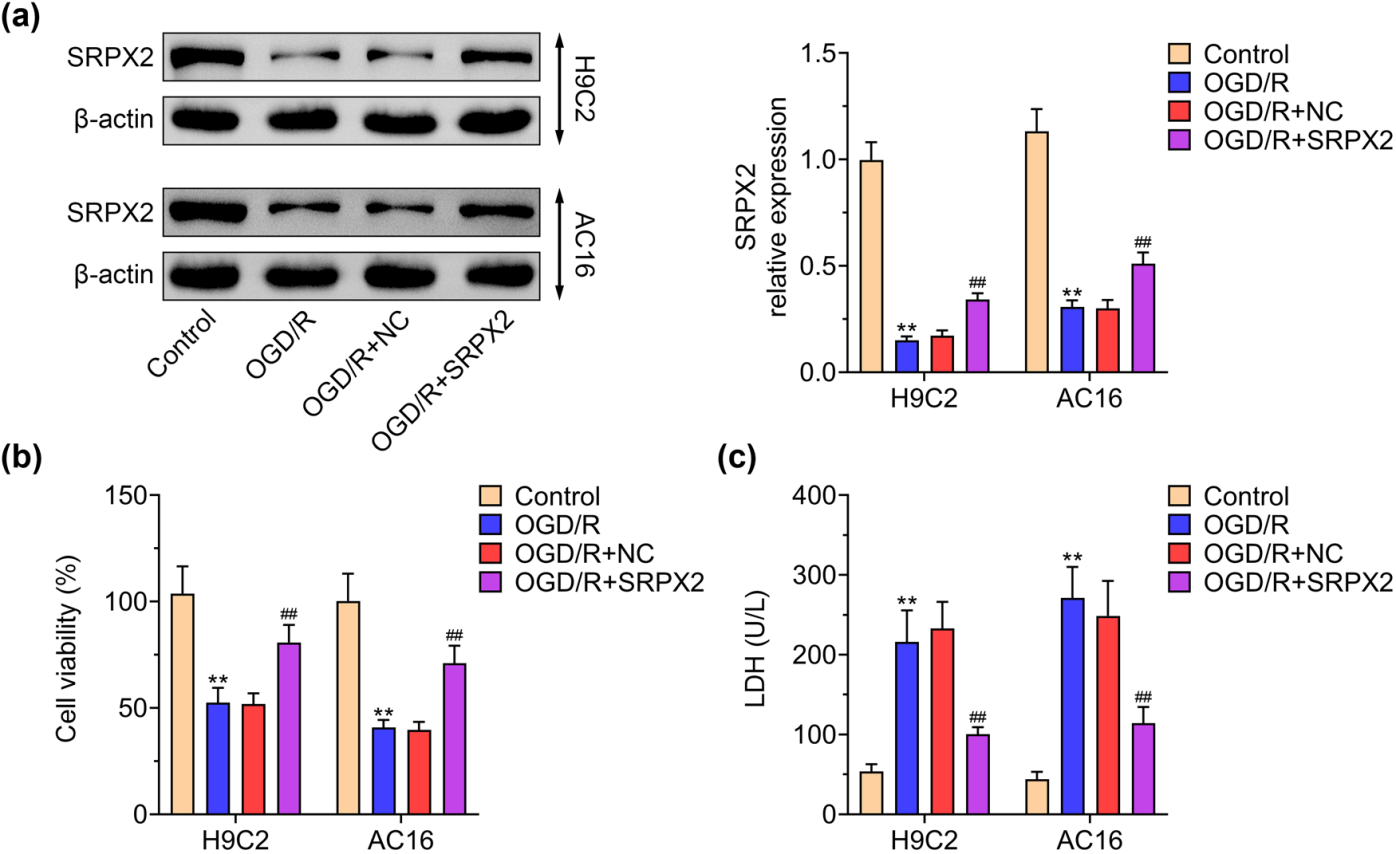
SRPX2 increases OGD/R-induced cell viability in H9C2 and AC16 cells: (a) expression of SRPX2 in OGD/R model and SRPX2 overexpression, (b) MTT assay detected the impact of SRPX2 on cell viability in H9C2 and AC16 cells with indicated treatment, and (c) LDH activity detected the impact of SRPX2 on cell viability in H9C2 and AC16 cells with indicated treatment. **, *p* < 0.01, ***, *p* < 0.001 vs control; #, *p* < 0.05, ##, *p* < 0.01, ###, *p* < 0.001 vs OGD/R.

After construction of OGD/R model in H9C2 and AC16 cells, we observed the downregulation of SRPX2 (Figure 1a). To reveal the role of SRPX2 in cell viability, MTT assay was performed. The cell viability after OGD/R was impaired and SRPX2 overexpression improved cell viability in H9C2 and AC16 cells (Figure 1b). The protective role of SRPX2 was evaluated by detecting the level of secreted LDH, an indicator of membrane cytotoxicity. The results showed that OGD/R conditioning significantly exacerbated the level of LDH, which were restored by SRPX2 overexpression (Figure 1c).

### 3.2 SRPX2 inhibits cell apoptosis challenged by OGD/R

**Figure 2:**
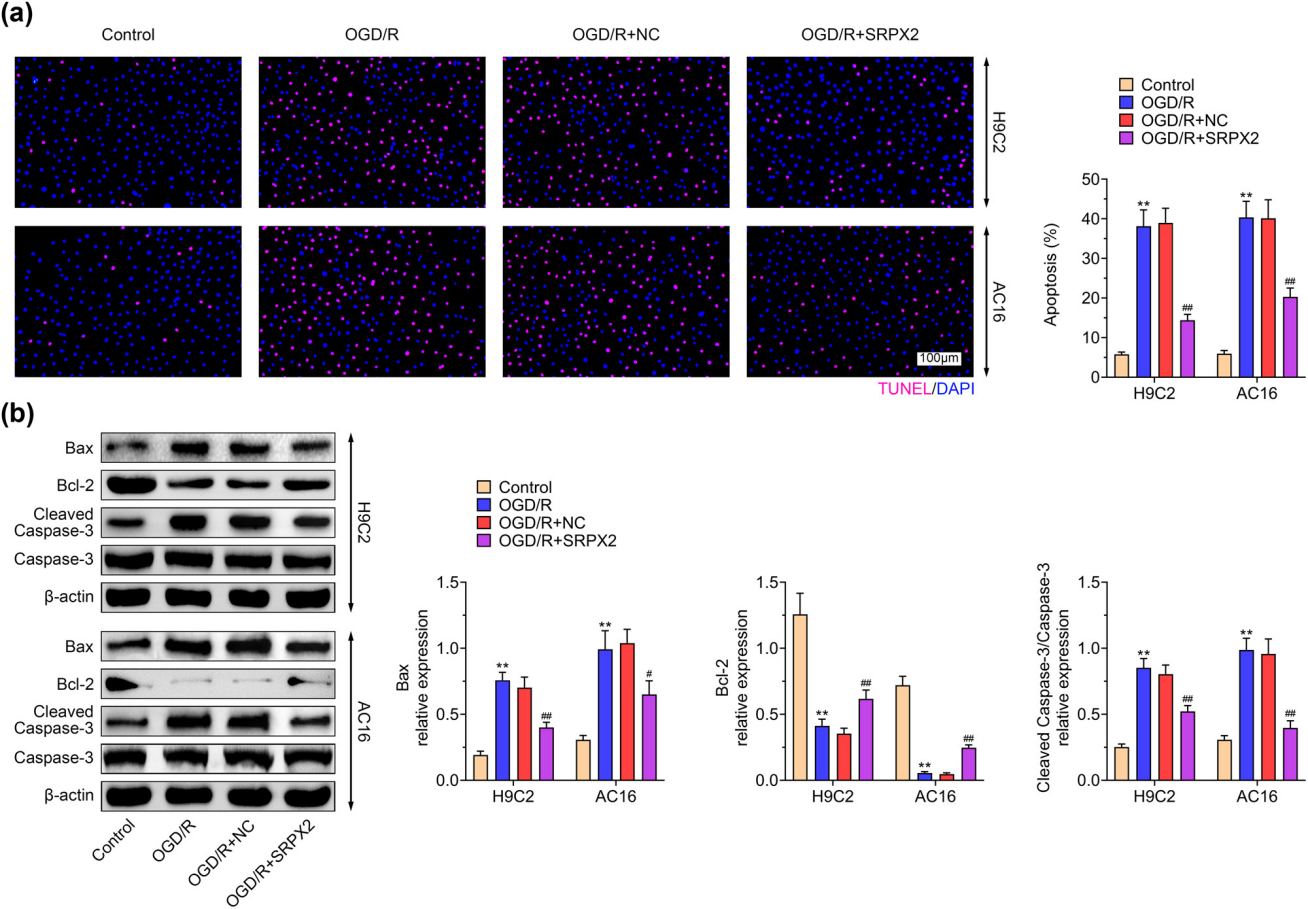
SRPX2 inhibits cell apoptosis challenged by OGD/R: (a) TUNEL assay of H9C2 and AC16 cells with indicated treatment and (b) level of apoptosis marker in H9C2 and AC16 cells with indicated treatment. **, *p* < 0.01, ***, *p* < 0.001 vs control; #, *p* < 0.05, ##, *p* < 0.01, ###, *p* < 0.001 vs OGD/R.

To reveal the effect of SRPX2 on cell apoptosis in OGD/R-stimulated H9C2 and AC16 cells, TUNEL assay was conducted. The TUNEL intensity and positive cells were accumulated in OGD/R treated cells and SRPX2 overexpression led to reduced TUNEL positive cells (Figure 2a). Consistently, the levels of Bax, andcleaved caspase-3 were reduced by SRPX2, while Bcl-2 was enhanced (Figure 2b), suggesting the effects on cell apoptosis. Therefore, SRPX2 regulates cell apoptosis in OGD/R-induced cells.

### 3.3 SRPX2 suppresses ER stress induced by OGD/R

**Figure 3:**
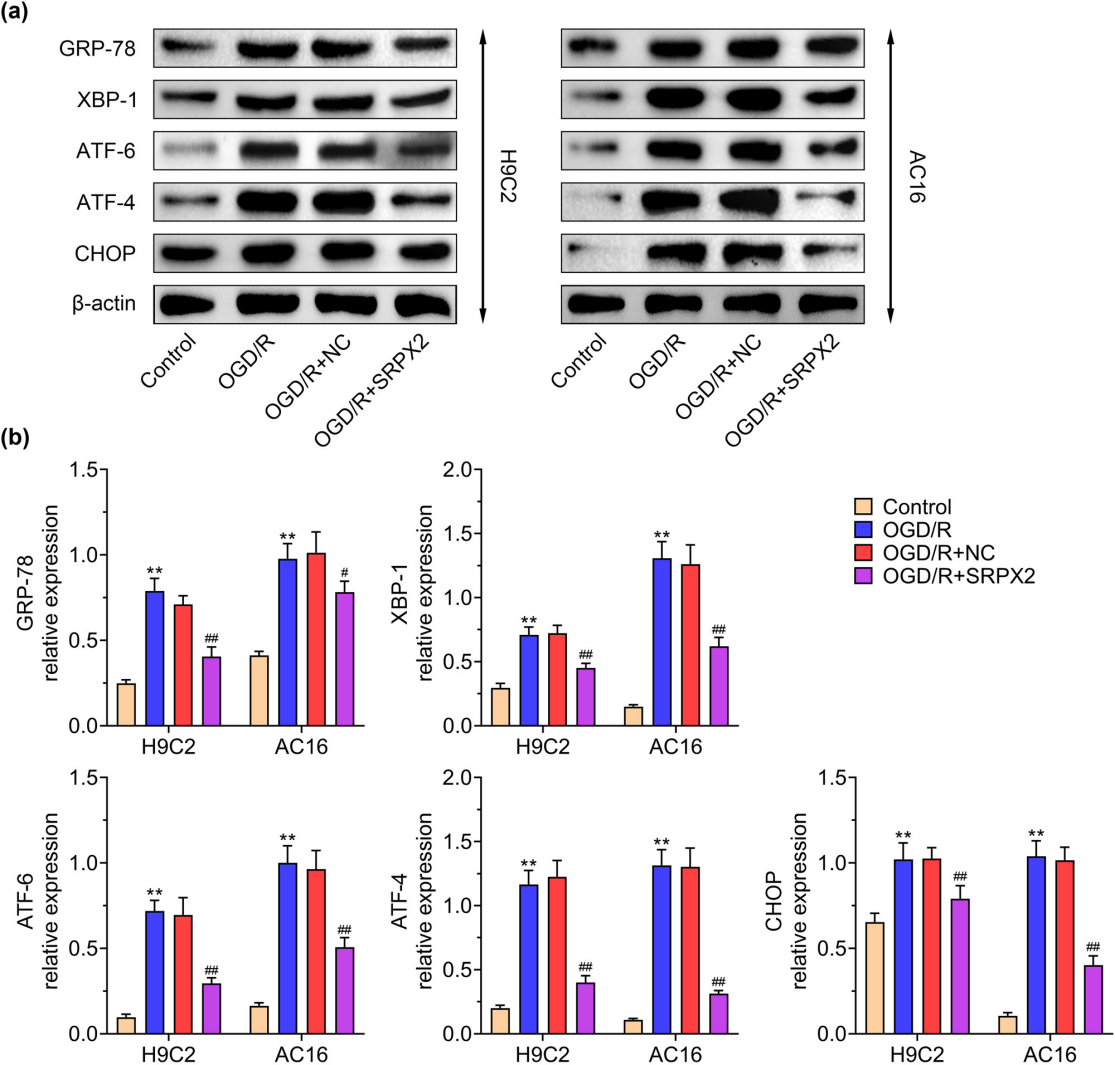
SRPX2 suppresses ER stress induced by OGD/R: (a and b) level of ER stress, GRP-78, XBP-1, ATF-6, ATF-4, CHOP in H9C2, and AC16 cells with indicated treatment. **, *p* < 0.01, ***, *p* < 0.001 vs control; #, *p* < 0.05, ##, *p* < 0.01, ###, *p* < 0.001 vs OGD/R.

ER stress plays an important role in the process of OGD/R. We examined the ER stress-related proteins in the OGD/R cells. The indicated markers of ER stress were upregulated by OGD/R and SRPX2 overexpression relieved the expression of these markers, including GRP78, XBP-1, ATF-6, ATF-4, and CHOP (Figure 3). Taken together, SRPX2 relieves ER stress induced by OGD/R injury.

### 3.4 SRPX2 protects OGD/R injury by activating the PI3K/Akt/mTOR pathway

**Figure 4:**
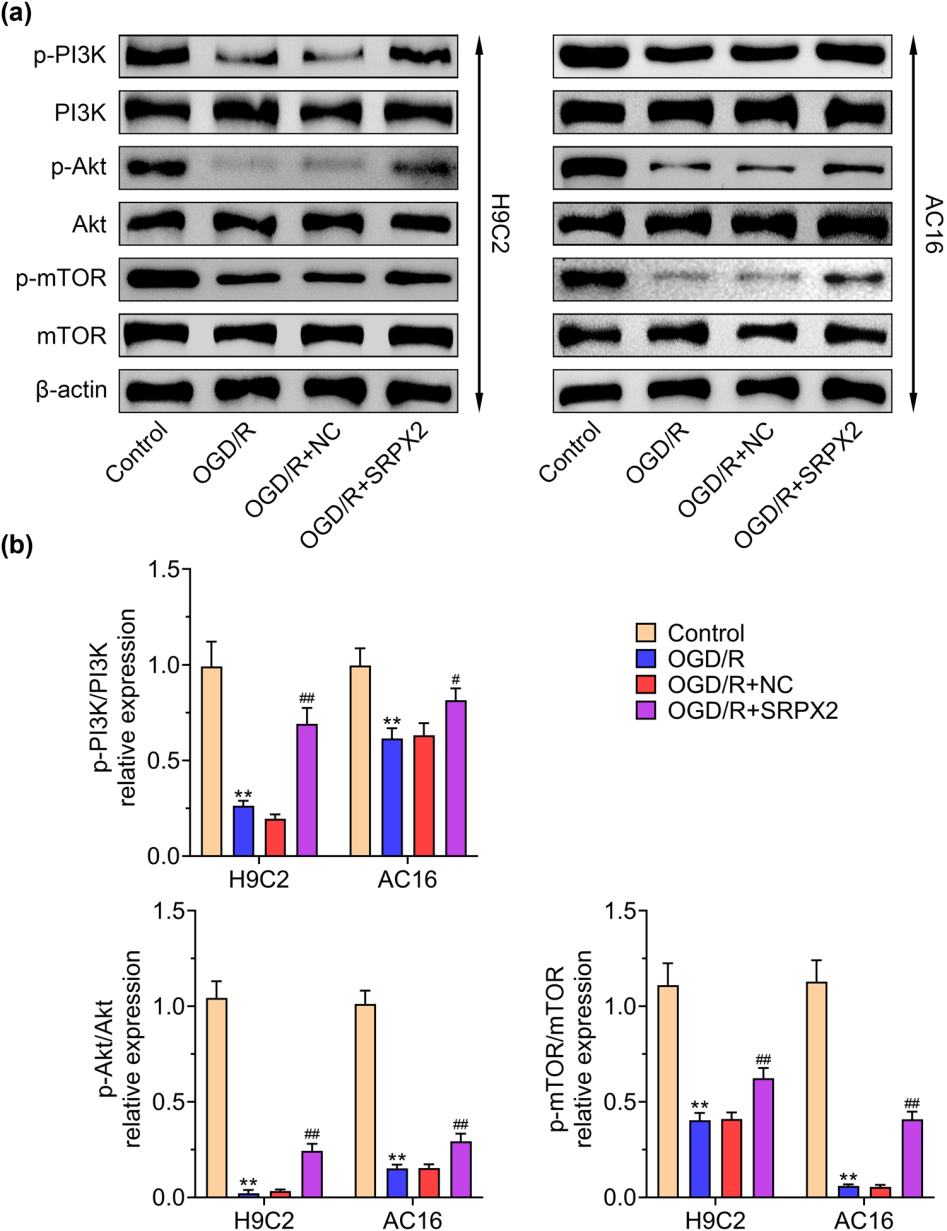
SRPX2 protects OGD/R injury by activating the PI3K/Akt/mTOR pathway: (a and b) expression of p-PI3K, p-Akt, and p-mTOR in H9C2 and AC16 cells with indicated treatment. **, *p* < 0.01, ***, *p* < 0.001 vs control; #, *p* < 0.05, ##, *p* < 0.01, ###, *p* < 0.001 vs OGD/R.

To explore the protective role of SRPX2 in OGD/R injury, the PI3K/Akt/mTOR signaling pathway was evaluated. OGD/R exposure repressed the expression of p-PI3K, p-Akt, and p-mTOR. SRPX2 induced the activation of PI3K/Akt/mTOR signaling pathway as indicated by the elevated level of p-PI3K, p-Akt, and p-mTOR, which was confirmed by immunoblot (Figure 4a and b). Taken together, SRPX2 protects OGD/R injury by activating the PI3K/Akt/mTOR pathway.

## 4 Discussion

MI is myocardial necrosis [19]. Clinically, the symptoms of MI are severe and cannot be completely relieved by rest and nitrates, accompanied by elevated serum myocardial enzyme activity and progressive electrocardiogram changes, and can be accompanied by arrhythmia [20,21]. For MI, timely rescue is the most important means to improve survival [22]. Myocardial ischemia-reperfusion injury is one of the main factors that restricts the optimal benefit of successful reperfusion therapy in patients with acute MI [19,23]. The mechanism of reperfusion injury remains unclear. Multiple studies explored the mechanism of MI via cell model. Herein, we found that SRPX2 could affect the progression of myocardial ischemia–reperfusion. We revealed that SRPX2 alleviated cardiomyocyte injury caused by oxygen and glucose deprivation reperfusion by improving endoplasmic reticulum stress (ERS)-induced apoptosis. Therefore, SRPX2 could serve as a promising target of MI.

In this study, we successfully constructed a cell model of myocardial ischemia–reperfusion through oxygen and glucose deprivation reperfusion. Subsequently, through qPCR, LDH release assay, and TTC staining, we revealed that SRPX2 was lowly expressed in IR rats and H9C2 cells induced by OGD/R. In addition, SRPX2 could increase the survival of H9C2 cells induced by OGD/R, which was confirmed through qPCR, MTT, and LDH release assay. Furthermore, SRPX2 could inhibit OGD/R-induced apoptosis and ER stress in H9C2 cells, confirmed by TUNEL and immunoblot assays. Therefore, these results suggested that SRPX2 could alleviate cardiomyocyte injury and MI. Importantly, TBI in rats induced a decrease in SRPX2 expression and the number of bilateral hypothalamic SRPX2 immunoreactive neurons [24]. Therefore, the decreased SRPX2 levels might be a candidate biomarker of hypothalamic injury [25]. In addition, SRPX2 was significantly downregulated in MI mice, suggesting its important role in MI pathogenesis.

In addition to its effects on MI, SRPX2 has been involved in multiple cellular processes. For example, SRPX2 could regulate the microglia-mediated synapse elimination during early development stage [26]. Its depletion suppressed the proliferation and the motility of prostate cancer cells via mediating PI3K/Akt/mTOR pathway. Similarly, we also found its effect on PI3K/Akt/mTOR pathway in H9C2 cells induced by OGD/R [27]. SRPX2 also mediated synapse formation and vocalization in mice.

Here, we also revealed the effects of SRPX2 on the ER stress. ERS can resist stress and protect cells by triggering the unfolded protein response signal transduction pathway and activating the expression of protective molecules such as ER chaperone [28]. If stress persists or is too strong, ERS will initiate the apoptosis process, thus promoting the progression of MI. Multiple studies have confirmed that ischemia–reperfusion injury can induce ERS, and ERS can stimulate many signal pathways in the cells [29]. In this study, we found that SRPX2 promoted ERS mainly through PI3K/Akt/mTOR pathway, and the precise molecular mechanism still needs to be further studied.

The antioxidant property of the SRPX2 has also been widely revealed. In fact, the chondroitin sulfate is a biomolecule which shows some antioxidant activity, and the antioxidant materials can affect the treatment and prevention of ischemia–reperfusion injuries [30–32]. Similarly, we confirmed it in our study; however, the mechanism needs further study. In addition, controlling the neuroprotective effects can prevent the ischemia–reperfusion, and the effect of SRPX2 on the neuroprotective effects also needs further study [33,34].

Importantly, the PI3K/Akt/mTOR pathway was reported to mediate the progression of MI. Several proteins affected the progression of MI and myocardial ischemia–reperfusion injury via this pathway [35]. Here we also noticed the effects of SRPX2 on this pathway, and revealed the mechanism underlying SRPX2 mediating myocardial ischemia–reperfusion progression. Therefore, the PI3K/Akt/mTOR pathway has the potential to serve as a target for MI treatment.

## 5 Conclusion

In summary, we investigated the role of SRPX2 in myocardial I/R. SRPX2 showed low expression in IR rats and H9C2 cells induced by OGD/R. SRPX2 could increase OGD/R-induced H9C2 cell survival. In addition, SRPX2 suppressed the apoptosis of OGD/R-induced H9C2 cells. Furthermore, we found that SRPX2 could inhibit ER stress induced by OGD/R in H9C2 cells. Mechanically, we found that SRPX2 suppressed the PI3K/Akt/mTOR pathway, and therefore attenuated OGD/R-induced injury in H9C2 cells. Therefore, SRPX2 could serve as a promising target for MI treatment.
